# Effects of Artemisinin on *Escherichia coli*–Induced Mastitis in Bovine Mammary Epithelial Cells and Mice

**DOI:** 10.3390/vetsci9080381

**Published:** 2022-07-26

**Authors:** Zhaoming Li, Jiaqing Hu, Xiaozhou Wang, Yongzhen Du, Jinhua Yin, Jian Gao, Bo Han, Shuai Cui, Yongxia Liu, Jianzhu Liu

**Affiliations:** 1 College of Veterinary Medicine, Shandong Agricultural University, Taian 271018, China; lizhaoming1210@126.com (Z.L.); hujiaqing0609@126.com (J.H.); sdau_wxz@163.com (X.W.); duyongzhen9873@163.com (Y.D.); 2 Research Center for Animal Disease Control Engineering, Shandong Agricultural University, Taian 271018, China; 3 College of Animal Science and Technology, Tarim University, Alar 843300, China; yinjinhuadky@126.com; 4 College of Veterinary Medicine, China Agricultural University, Beijing 100193, China; gaojian2016@cau.edu.cn (J.G.); hanbo@cau.edu.cn (B.H.); 5 Modern Animal Husbandry Development Service Center of Dongying City, Dongying 257091, China; cuishuai@dy.shandong.cn

**Keywords:** mastitis, *E. coli*, artemisinin, MAC-T cells, mice

## Abstract

**Simple Summary:**

Bovine mastitis is a persistent and inflammatory reaction of the udder tissue that is usually caused by microbial infection, which can result in substantial losses due to reduced milk yield. *Escherichia coli* is considered a causative environmental pathogen and has been reported as a common cause of bovine mastitis worldwide. Because of its pathogenicity, *Escherichia coli* is always an important problem to the dairy industry worldwide and also poses a threat to food safety and public health, and with the widespread use of antibiotics, the resistance of *Escherichia coli* is increasing. Despite considerable research on bovine mastitis, the disease still remains one of the most prevalent and costly diseases of the dairy industry. The need to control mastitis is driven by multiple considerations, including milk quality, reductions in antimicrobial use, and animal welfare. Artemisinin is an antimalarial drug that was developed from a Chinese traditional herb, Qinghao. In recent years, other effects of artemisinin (including antitumor, anti-inflammatory, antifungal, etc.) have been increasingly discovered and applied. In this study, we demonstrated that artemisinin possesses a protective effect toward *Escherichia coli*–induced mastitis, thus providing a practical approach for the clinical control of mastitis.

**Abstract:**

Bovine mastitis is an important disease affecting dairy farming, and it causes large economic losses to the dairy industry. *Escherichia coli* (*E. coli*) is considered to be a causative environmental pathogen and frequently enters into mammary glands, causing inflammation. Artemisinin is a highly effective malaria remedy and is not easy to develop drug resistance to. In recent years, other effects of artemisinin (including antitumor, anti-inflammatory, antifungal, etc.) have been increasingly discovered and applied. The current study aimed to investigate whether artemisinin could attenuate *E. coli*–induced inflammation. Through the *E. coli* mastitis model in MAC-T cells and mice, the protective effects of artemisinin were analyzed by CCK-8 (Cell Counting Kit-8), Western blot, and RT-qPCR. The results showed that artemisinin reversed the decrease of cell viability and upregulated TLR4 (toll-like receptor 4)/NF-κB (nuclear factor κB) and MAPK (mitogen activated protein kinase)/p38 signaling pathways, as well as restrained the expression of TNF-α, IL-6, and IL-1β mRNA caused by *E. coli*. Meanwhile, artemisinin also alleviated mammary tissue damage, reduced inflammatory cells’ infiltration, and decreased the levels of inflammatory factors in a mice mastitis model. This study demonstrated that artemisinin alleviated the inflammatory response of mouse mastitis and MAC-T cells induced by *E. coli*, thus providing a practical approach for the clinical control of mastitis.

## 1. Introduction

Bovine mastitis is a mammary tissue inflammatory disease that is caused by mechanical irritation, pathogenic microorganisms, and chemical and physical damage, and it is among the most common diseases in dairy farms. It not only reduces milk production but increases treatment costs, results in a loss of feed utilization, and creates milk waste [1]. The clinical manifestations of bovine mastitis are usually redness, swelling, fever, and pain in the mammary glands [2].

The defense machinery of mammary glands can be classified into nonspecific and specific immunity. Nonspecific immunity, also called innate immunity, is the main defense mechanism in the early phases of infection [3]. The specific immune system can facilitate or selectively eliminate pathogens by specifically recognizing their pathogenic factors through antibody molecules, macrophages, and lymphocytes [3,4,5,6]. In the mammary gland, innate and specific immunity coordinate with each other to protect against diseases. If the treatment is not timely or thorough, it is difficult to recover, and there is a risk of recurrence [7,8].

*E. coli* is widespread in the natural environment and does not cause infection in humans and animals under normal circumstances, but results in illness when the immunity of humans and animals is poor [9]. In dairy farms, *E. coli* is the most frequently occurring etiologic agent of environmental mastitis, and its incidence is closely related to the cows’ age, lactation period, and immune status, and other factors [10]. The pathogenicity of *E. coli* is determined by a variety of virulence factors, including pilin, adhesins, and lipopolysaccharides. To date, no specific virulence factors involving only bovine mastitis caused by *E. coli* have been identified [11]. After invading the host, *E. coli* will bind to TLR4 to activate the host’s innate immune system, thus inducing the activation of the MAPK and NF-κB signaling pathways [12]. The sensing of *E. coli* in bovine mammary glands involves epithelial cells that trigger a cascade of immunity-related processes [13]. Furthermore, a recent study suggested that the pathogenicity of *E. coli* in bovine mammary glands is associated with the presence of a new pathogenic phenotype known as mammary pathogenic *Escherichia coli* (MPEC) [14]. Meanwhile, the increasing antibiotic resistance of bacteria is one of the reasons for the low cure rate of mastitis in dairy cows and has attracted wide attention and intensive research in the livestock and public health industries [15,16,17,18].

Artemisinin is an endoperoxide terpene lactone compound that is found mainly in the Chinese medicine Artemisia annua [19,20]. Its derivatives include dihydroartemisinin, artesunate, artemisinin methyl ether, and others. In addition to its anti-malarial effects, artemisinin also has various biological functions in terms of antioxidant, anti-inflammatory, and vascular protection [21,22,23,24]. Wang et al. found that artemisinin treatment inhibited the expression of NF-κB-pathway-related proteins and the release of inflammatory factors such as IL-6 induced by LPS or *E. coli*, thus reducing the mortality of mice infected with *E. coli* [25]. These findings support the therapeutic potential of artemisinin for mastitis.

Despite accumulating evidence that artemisinin is effective in suppressing inflammation-related diseases, studies related to the treatment of bovine mastitis are lacking. Whether artemisinin could alleviate *E. coli*–induced bovine mastitis is uncertain. Therefore, this study established a mouse mastitis model by injecting *E. coli* into mammary glands and infecting MAC-T cells with *E. coli* to investigate whether artemisinin exerts anti-inflammatory defense effects by regulating the expression of inflammatory-pathway-related proteins, as well as inflammatory factors.

## 2. Materials and Methods

### 2.1. Reagents and Antibodies

Artemisinin was provided by Solarbio (Beijing, China). DMEM/high-glucose medium was obtained from Servicebio (Wuhan, China). The following primary antibodies were used: NF-κB p-65 (1:1000) and phosphorylated NF-κB p-65 (1:700) were procured from ABclonal (Wuhan, China); p-38 (1:1000), phosphorylated p38 (1:700), IKK (1:1000), and phosphorylated IKK (1:700) were purchased from CST (Boston, MA, USA); and TLR4 (1:1000), Myd88 (1:1000), β-actin (1:1000), GAPDH (1:80000), and Tubulin (1:50000) were purchased from Proteintech (Wuhan, China). The secondary antibodies, HRP-conjugated Affinipure Goat Anti-Rabbit IgG (1:5000) and HRP-conjugated Affinipure Goat Anti-Mouse IgG (1:5000), were obtained by Proteintech (Wuhan, China). The RT-qPCR-related reagents were provided by Accurate Biotechnology (Hunan, China).

### 2.2. Bacteria Strains and Culture

*E. coli* (ATCC25922) was inoculated on LB agar and incubated in an incubator at 37 °C. A randomly selected single colony was added to the LB broth and placed in a shaker at 37 °C and 200 rpm. After 12 h, the OD_600nm_ values were measured.

### 2.3. Cell Culture

MAC-T (bovine mammary alveolar cell-T) cells were cultured in DMEM/high-glycemic medium containing 5% fetal bovine serum and grown in a sterile incubator at 37 °C, containing 5% CO_2_. When the cell fusion reached 90%, the following experiments were performed.

### 2.4. Mastitis Mouse Model and Sample Collection

Compared with other experimental animals, the mouse mastitis model is considered to be a straightforward and suitable model to study bovine mastitis because of its ease of manipulation and lower cost; it provides valuable information about the pathogenic mechanisms of bovine mastitis [26]. SPF Kunming mice were housed at 25 °C, 50% humidity, with 12 h of light and 12 h of darkness, in an experimental animal housing, and provided with food and water. The mice used in this experiment met the requirements of animal care and use suggested by the Committee of Shandong Agricultural University (SDAUA-2021-008). The model of mouse mastitis was established according to the previously described methods [27,28]. Female mice with similar delivery time were distributed to four groups randomly: control group, artemisinin control group, *E. coli* group, and artemisinin treatment group, with ten mice in each group. The female mice were slowly injected with 10 μL of 1 × 10^7^ CFU/mL *E. coli* solution into the fourth pair of mammary glands, using a microsyringe once daily for three days. The female mice in the artemisinin-treatment group were the same as the *E. coli* group and treated with 50 mg/kg of artemisinin by oral gavage once daily for three days after the onset of mastitis was induced in the mice. Serum and mammary tissue were collected from the mice at the end of treatment.

Mice were anesthetized, and the fourth pair of mammary tissues was collected to make pathological sections and observed for pathological damage. The collected blood of mice was left for 2 h and then centrifuged at 3000 rpm for 5 min to obtain the serum, which was then kept at −80 °C for backup.

### 2.5. Cell Viability Assay

MAC-T cells were cultured in 96-well plates at a density of 1 × 10^4^ cells per well in an incubator. MAC-T cells were infected with different concentrations (10^5^, 10^6^, 10^7^, and 10^8^ CFU/mL) of *E. coli* for 4, 6, and 8 h, with or without artemisinin (100 μg/mL) treatment. Cell viability was measured by using the CCK-8 (Cell Counting Kit-8) method. Briefly, 10 μL CCK-8 reagent was added to cells and incubated for 2 h. The absorbance was detected at 450 nm. Cell viability = (experimental group − blank group)/(control group − blank group) × 100%; the blank group contained medium and CCK-8 reagent but did not contain cells and *E. coli*, and the control group contained cells, medium, and CCK-8 reagent but did not contain *E. coli*. The results were obtained from three independent experiments. After combining the results of this part, MAC-T cells infected with *E. coli* of 10^7^ CFU/mL for 4 h were selected to perform the subsequent experiments.

### 2.6. Quantitative Real-Time PCR (RT-qPCR)

MAC-T cells were infected with 10^7^ CFU/mL of *E. coli* for 4 h, with or without artemisinin (100 or 200 μg/mL) treatment. The cells in each group were lysed with pre-cooled TRIzol and chloroform and left to stand for 5 min. The cell lysate was centrifuged at 12,000 g for 10 min. The supernatant was obtained and added to isopropanol and centrifuged again. Then the RNA precipitate was solubilized by RNA-free water. The cDNA was synthesized according to the kit operation, and IL-6, IL-1β, and TNF-α mRNA expressions were assayed by qPCR. The primers used in this study are shown in Table 1.

### 2.7. Western Blot

MAC-T cells were infected with 10^7^ CFU/mL of *E. coli* for 4 h, with or without artemisinin (100 or 200 μg/mL) treatment. The protein lysis solution and protease inhibitor were added to the cells for lysis. The lysed cells were collected into a 1.5 mL centrifuge tube, using a cell scraper. After centrifugation, the supernatant was obtained and transferred to a new centrifuge tube. The protein concentration was measured by using the BCA method, run with 10% sodium dodecyl sulfate polyacrylamide gels, and then electo-transferred to PVDF membranes. After PVDF membranes were blocked with 5% BSA solution at room temperature for 2 h, the membranes were incubated with the primary antibody overnight at 4 °C. After that, the membranes were incubated with secondary antibody at room temperature for 1 h. After washing with TBST, the aim target proteins were visualized by using ECL Western Detection Reagent and analyzed by using ImageJ software.

### 2.8. Histopathological Examination

The fresh tissues were soaked in 4% formalin fixative; after 72 h, the tissues were soaked in gradient alcohol for dehydration. Paraffin sections were prepared for HE (hematoxylin–eosin) staining, and once stained, the stained sections were dehydrated with anhydrous ethanol, washed with xylene, and placed in a ventilation cabinet. Finally, the slices were covered with neutral resin and observed under an optical microscope. 

### 2.9. Statistical Analysis

The results of the presented experiments were obtained from three independent experiments and presented as mean ± standard deviation (mean ± SD). The statistical significance of differences between groups was analyzed by One-Way ANOVA, followed by Tukey’s post hoc test. Experimental data were analyzed by using SPSS biostatistics software Version 24.0 and charted by using Graph Pad Prism. The *p*-values less than 0.05 were regarded as significant.

## 3. Results

### 3.1. Artemisinin Reverses the Decrease of Cell Viability in E. coli–Infected MAC-T Cells

**Figure 1:**
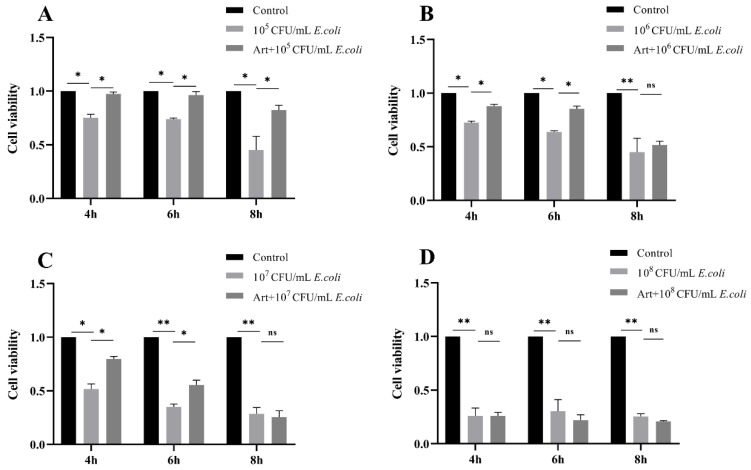
(**A**–**D**) Effects of different concentrations of *E. coli* on MAC-T cells’ activity after 4, 6, and 8 h of infection and effect of artemisinin on the activity of *E. coli*–infected MAC-T cells. * *p* < 0.05, and ** *p* < 0.01.

Firstly, no effect on cell viability and *E. col*i proliferation was detected that when MAC-T cells and *E. coli* exposed to artemisinin alone. To further explore the effect of artemisinin on cells challenged by *E. coli*, MAC-T cells were exposed to different concentrations of *E. coli* for 4, 6, and 8 h, with or without artemisinin treatment. As shown in Figure 1, the viability of MAC-T cells was significantly reduced in the *E. coli*–infected group as compared with the control group. Artemisinin treatment markedly inhibited the decrease in cell viability caused by 10^5^ CFU/mL *E. coli* infection for 4, 6, and 8 h; and 10^6^ and 10^7^ CFU/mL *E. coli* infection for 4 and 6 h. After treating the MAC-T cells for 4 h with 10^7^ CFU/mL *E. coli*, cell viability decreased by about 50%. In this study, we focused on the immunostimulatory effect on host cells at the early stage of *E. coli* infection, and, therefore, the MAC-T cells infected with *E. coli* of 10^7^ CFU/mL for 4 h were selected to perform the subsequent experiments.

### 3.2. Artemisinin Inhibits the Expression of TLR4/NF-κB Inflammatory Pathway

**Figure 2:**
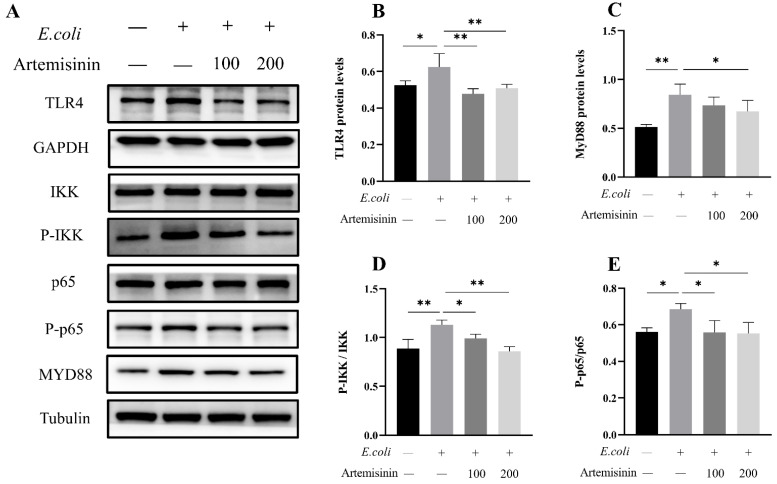
(**A**–**E**) Protein levels of TLR4, MyD88, p65, p-p65, IKK, and p-IKK were determined by Western blot. GAPDH and Tubulin were used as loading controls. Note that “−” and “+” indicate not added and added, respectively. * *p* < 0.05, and ** *p* < 0.01.

In the present study, *E. coli* dramatically induced the expression of TLR4 and the adaptor protein Myd88 in MAC-T cells, whereas artemisinin treatment significantly reversed this change. Next, the phosphorylation of IKK and NF-κB/p65 was examined, and the findings indicate that artemisinin can significantly reduce *E. coli*–induced phosphorylation of IKK and NF-κB/p65 (Figure 2). These results indicate that artemisinin inhibited *E. coli*–induced upregulation of the TLR4/NF-κB signaling pathway.

### 3.3. Artemisinin Inhibits the Activation of MAPK/p38 Inflammatory Pathway

**Figure 3:**
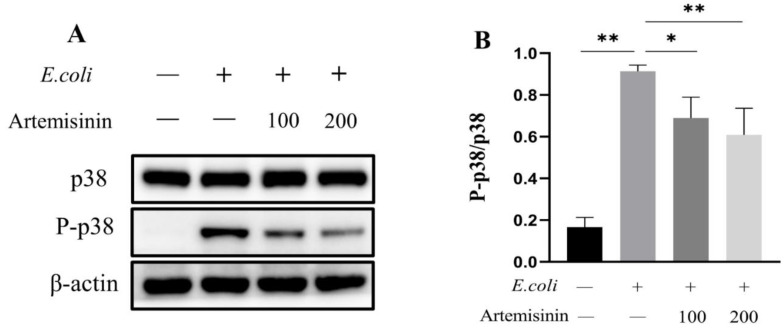
The changes of MAPK/p38. (**A**) Western blot. β-actin served as the control. (**B**) Statistical analysis of MAPK/p38 changes. * *p* < 0.05, and ** *p* < 0.01.

It is well-known that MAPK/p38 is also an important inflammatory pathway. Therefore, in this study, the changes of p38 protein were examined in *E. coli*–infected MAC-T cells. As shown in Figure 3, *E. coli* infection significantly promoted the phosphorylation of p38 in MAC-T cells, while artemisinin remarkably inhibited this effect. This suggests that artemisinin may inhibit the inflammatory response through MAPK/p38.

### 3.4. Artemisinin Reduces IL-1β, IL-6, and TNF-α mRNA Expression in E. coli–Induced MAC-T Cells

**Figure 4:**
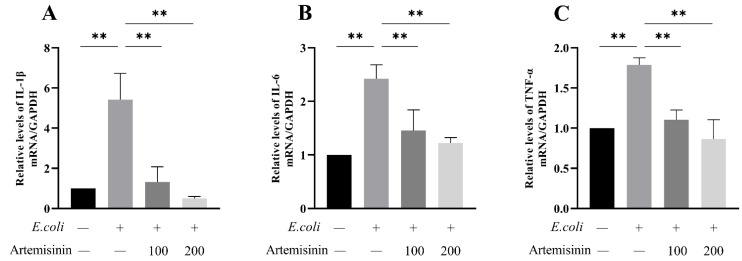
RT-qPCR to measure inflammatory factor expression levels: (**A**) IL-1β, (**B**) IL-6, and (**C**) TNF-α. GAPDH as control. ** *p* < 0.01.

Inflammatory factors could contribute to the incidence and progression of inflammation, such as IL-1β, IL-6, and TNF-α. As seen in Figure 4, the expression of mRNA for these three inflammatory factors was obviously raised after *E. coli* infection; meanwhile, artemisinin treatment effectively inhibited the expression of these inflammatory factors. This demonstrates that artemisinin could alleviate the MAC-T cells’ response to inflammation elicited by *E. coli*.

### 3.5. Artemisinin Reduces Serum Levels of IL-1β, IL-6 and TNF-α in Mice

**Figure 5:**
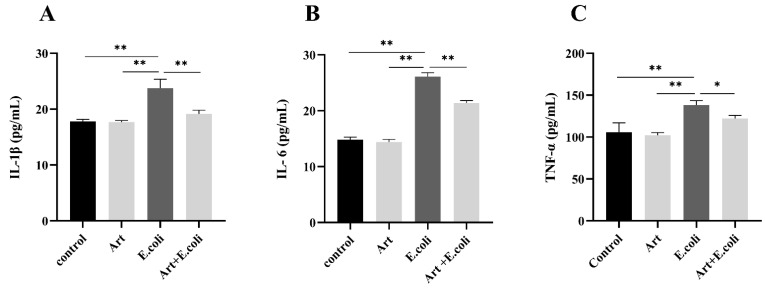
ELISA to measure the level of inflammatory factors in mouse serum: (**A**) IL-1β, (**B**) IL-6, and (**C**) TNF-α. * *p* < 0.05, and ** *p* < 0.01.

The levels of IL-6, IL-1β, and TNF-α in mice serum were measured by using ELISA. The results shown in Figure 5 revealed that artemisinin suppressed the elevated levels of IL-1β, IL-6, and TNF-α in the serum of mice caused by *E. coli*.

### 3.6. Artemisinin Relieves the Pathological Damage of Mammary Gland in E. coli–Induced Mastitis Mice

**Figure 6:**
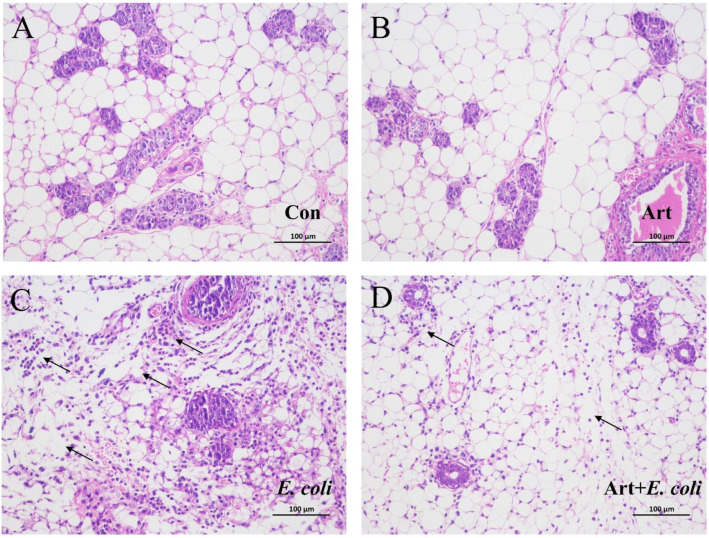
Effects of artemisinin on the histology of *E. coli*–induced mammary gland pathology in mice (×200): (**A**) control group, (**B**) artemisinin control group, (**C**) *E. coli* group, and (**D**) artemisinin treatment group. Arrows indicates the infiltrated inflammatory cells.

To further evaluate the protective effect of artemisinin on mastitis, the mouse model was established by mammary duct infusion of *E. coli*. As illustrated in Figure 6, compared to the control group, the mice in the *E. coli* group had markedly more inflammatory cells that had infiltrated the mammary acini, thickened alveolar walls, and disrupted alveolar lumen. The artemisinin treatment significantly alleviated the tissue damage caused by *E. coli*, the inflammatory infiltrate was reduced, and the mammary acini tended to be intact.

## 4. Discussion

*E. coli* is widely distributed in the environment and often invades the udder tissues of dairy cows, causing inflammation. It is considered to be one of the primary causative agents of mastitis. Due to the widespread use of antibiotics, *E. coli* resistance has gradually increased [29]. *E. coli*–induced mastitis has not only seriously affected the sustainable development of dairy farming and the dairy industry but has also brought considerable hidden danger to public health safety and food safety [30,31]. Artemisinin is an important antimalarial drug that was developed from a Chinese traditional herb, Qinghao [19]. With the development of the research, other effects of artemisinin, which is very effective against malaria, such as anti-inflammatory and antiviral, are gradually being discovered [21]. Zhang et al. evaluated the anti-inflammatory effects of artemisinin in mouse models stimulated by LPS [32]. Qiao et al. showed that artemisinin could inhibit TLR4 signaling and inflammatory responses in LPS-induced BV2 microglial cells [33]. Kim et al. demonstrated that artemisinin has anti-inflammatory activities against periodontopathic bacteria [34]. Whether artemisinin could exert protective effects against *E. coli*–induced inflammation remains to be elucidated. In the present study, we explored the effect of artemisinin on *E. coli*–induced mastitis and studied the related inflammatory signaling pathways, and this helped us to further understand whether artemisinin could be an alternative to antibiotics for the prophylaxis and therapy of mastitis.

*E. coli* is the environmental pathogen that causes bovine mastitis. After milking during lactation, when the sphincter around the teat duct is in a relaxed state, or when prolonged milking changes the teat state, *E. coli* enters the milk pool along the teat duct, and the milk provides an ideal environment for *E. coli* to multiply [35]. The pathogenicity of *E. coli* is closely related to its own adhesion and aggressiveness, as well as the age and immunity of the cow. It has previously been shown that it takes only 15.2 min for *E. coli* to multiply to 10^9^ CFU/mL in milk, and the faster *E. coli* multiplies, the greater the number of virulence factors, such as LPS, in the mammary gland, and the greater the damage caused. Continuous infection with *E. coli* may be an essential reason for mammary gland damage in dairy cows [36,37].

Artemisinin and its derivatives have shown potent efficacy in malaria, but as research progresses, its anti-inflammatory aspects are also being reported. In this study, artemisinin inhibited the activation of TLR4/NF-κB pathways in *E. coli*–infected MAC-T cells, and this may be a potential mechanism for artemisinin treatment of mastitis. The effects of artemisinin on inflammatory diseases may be reflected in the modulation of inflammatory pathways. Zhang et al. found that artemisinin increased the activity of porcine mammary epithelial cells after LPS stimulation and attenuated LPS damage to porcine mammary glands by inhibiting NF-κB and MAPK inflammatory pathways [38]. Previous studies have shown that, when mice were attacked by heat-inactivated *Staphylococcus aureus*, artemisinin improved survival by suppressing TLR2 expression and activation of NF-κB, and exerted a protective effect in a dose-dependent manner. In an in vitro assay, artemisinin inhibited the release of TNF-α from *S. aureus* or peptidoglycan-induced mouse peritoneal macrophages [39]. Yuan et al. discovered that artemisinin inhibited neutrophil infiltration in rosacea-like mice and suppressed the activation of the NF-κB pathway, thus suggesting that artemisinin may improve chronic inflammatory skin diseases of the face by modulating immune response and angiogenesis [40]. A growing number of studies have indicated the potential of artemisinin in the treatment of inflammation [25,41,42]. 

The TLR4/NF-κB pathway has a critical function in the regulation of inflammatory responses. The cell membrane receptor TLR4 recognizes exogenous stimuli such as lipopolysaccharide from *E. coli*, and the signal is transmitted through MyD88, activates NF-κB, and then modulates many of the pro-inflammatory cytokines and chemokine transcripts that contribute to the development of mastitis. Previous studies have demonstrated that mRNA expression of TLR4 was upregulated and levels of inflammatory factors such as IL-8 and IL-6 were increased in MAC-T cells after *E. coli* infestation [43,44]. LPS stimulation of TLR4 induces activated NF-κB and MAPK inflammatory pathways [45]. These findings are similar to those of our study. Furthermore, it was demonstrated that *E. coli* infection mediated the inflammatory response through activation of NLRP3 and NLRC4 inflammasome in bovine mammary epithelial cells. The interaction of LPS with TLR4 resulted in the activation of NLRP3 and subsequent formation of NLRP3 inflammasome with ASC proteins. Inflammasome induced caspase-1 shearing and the production of active IL-1β and IL-18 [46,47]. It remains to be investigated whether artemisinin could alleviate *E. coli*–induced mastitis by inhibiting the activation of inflammasome in MAC-T cells.

In the present study, *E. coli* significantly upregulated the levels of inflammatory factors in MAC-T cells and mouse serum, but this was markedly reversed by artemisinin with a dose-dependent manner. The MAPK/p38 inflammatory pathway can be activated by many inflammation-related stimuli and has a key function in modulating the biological synthesis of pro-inflammatory cytokines [48]. It represents a therapeutic target for many inflammatory diseases [49]. In addition, pro-inflammatory cytokines serve as direct inflammatory indicators designed to reflect the severity of inflammation [50]. We examined the mRNA expression levels of IL-1β, IL-6, and TNF-α, using RT-qPCR, and found they were all remarkably elevated in cells infected by *E. coli*, whereas the intervention of artemisinin resulted in a considerable decrease in the expression of IL-1β, IL-6, and TNF-α that further suggests that artemisinin could alleviate *E. coli*–induced inflammation reactions. 

The papillae and papillary ducts are the important line of defense for the mammary glands. When the natural barrier fails, the pathogens enter the milk pool along the nipple ducts. As the bacteria multiply, the inflammatory cells in the mammary tissue also gradually increase, further aggravating the damage and the loss of structural integrity of the mammary acini [35,51,52]. To further understand the protective effect of artemisinin on *E. coli*–induced bovine mastitis, an *E. coli*–induced mastitis model in mice was established. Our results showed that artemisinin treatment could obviously reduce inflammatory cells’ infiltration, alleviate mammary tissue damage, and inhibit the increase of inflammatory factors in the serum of *E. coli*–induced mice.

## 5. Conclusions

In conclusion, this study demonstrates that artemisinin possesses a protective effect toward *E. coli*–induced mastitis, thus providing an approach for treatment of bovine mastitis.

## Figures and Tables

**Table 1:** Primers used in this study.

Primers Name	Primers Sequence (5′→3′)
GAPDH	F: GATGGTGAAGGTCGGAGTGAAC
	R: GTCATTGATGGCGACGATGT
IL-1β	F: CCTATTCTCTCCAGCCAACCT
	R: CTCATTCTCGTCACTGTAGTAAGC
IL-6	F: GGACTACCTCCAGAACGAGTATGA
	R: TCTTCTCCAGCAGGTCAGTGT
TNF-α	F: GCCCTCTGGTTCAAACACTCA
	R: CGGAGAGTTGATGTCGGCTAC

## Data Availability

Not applicable.
